# A phase II trial of goserelin (Zoladex) in relapsed epithelial ovarian cancer.

**DOI:** 10.1038/bjc.1992.126

**Published:** 1992-04

**Authors:** M. J. Lind, B. M. Cantwell, M. J. Millward, A. Robinson, M. Proctor, D. Simmons, J. Carmichael, A. L. Harris

**Affiliations:** University Department of Clinical Oncology, Newcastle General Hospital, Newcastle upon Tyne, UK.

## Abstract

Thirty patients with advanced epithelial ovarian cancer were treated with the luteinising hormone releasing agonist, goserelin. There were two partial responses lasting 40 and 105 weeks respectively. In addition five patients had disease stabilisation lasting 25, 35, 40, 66 and 70 weeks respectively and 23 patients had progressive disease. No significant or unexpected toxicities occurred. This minimally toxic therapy halted disease progression for 6 months or more in 23% of patients, the majority of whom were heavily pretreated. There were five early deaths due to disease progression. The use of goserelin in patients with epithelial ovarian cancers resistant to or relapsing soon after first line platinum based chemotherapy needs to be further evaluated.


Br. J. Cancer (1992), **65**, 621–623 © Macmillan Press Ltd., 1992

### A phase II trial of goserelin (Zoladex) in relapsed epithelial ovarian cancer

M.J. Lind[1], B.M.J. Cantwell[1], M.J. Millward[1], A. Robinson[1], M. Proctor[1], D. Simmons[1], J. Carmichael[2] & A.L. Harris[2]

[1]*University Department of Clinical Oncology, Regional Radiotherapy Centre, Newcastle General Hospital, Westgate Road, Newcastle upon Tyne NE4 6BE;* [2]*ICRF Clinical Oncology Unit, Churchill Hospital, Oxford OX3 7LF, UK.*

**Summary** Thirty patients with advanced epithelial ovarian cancer were treated with the luteinising hormone releasing agonist, goserelin. There were two partial responses lasting 40 and 105 weeks respectively. In addition five patients had disease stabilisation lasting 25, 35, 40, 66 and 70 weeks respectively and 23 patients had progressive disease. No significant or unexpected toxicities occurred. This minimally toxic therapy halted disease progression for 6 months or more in 23% of patients, the majority of whom were heavily pretreated. There were five early deaths due to disease progression. The use of goserelin in patients with epithelial ovarian cancers resistant to or relapsing soon after first line platinum based chemotherapy needs to be further evaluated.

Despite the high response rates achieved with either single agent platinum chemotherapy or platinum based combination chemotherapy in women with advanced epithelial ovarian cancer, long term survival in this disease remains poor with the majority of patients ultimately relapsing and dying of their disease (Wharton *et al.*, 1984). Thus there is a need to improve chemotherapy for both initial therapy and for relapsed ovarian cancer. Furthermore there is the need to identify novel therapies for patients with a low probability of responding to conventional cytotoxic chemotherapy (Blackledge *et al.*, 1989).

Several studies have demonstrated the presence of both sex steroid hormone receptors and gonadotrophin releasing hormone (GnRHs) receptors in epithelial ovarian tumour cells. It has been demonstrated that 50% of epithelial ovarian cancers are positive for oestrogen receptors, whilst 53% are progesterone receptor positive and 88% are androgen receptor positive (Rao *et al.*, 1990). Furthermore some 80% of epithelial ovarian cancers possess low affinity, high capacity binding sites for gonadotrophin releasing hormone (Emons *et al.*, 1989). Langdon *et al.* (1990) have demonstrated that 17 $\beta$ oestradiol will stimulate the growth of oestrogen receptor positive human ovarian carcinoma cell lines and that tamoxifen will inhibit this oestrogen stimulated growth. The functional *in vivo* importance of this range of receptor expression is uncertain and the use of hormone manipulations, such as tamoxifen, have yielded variable and generally poor clinical responses (Slotman & Rao, 1988).

Goserelin (Zoladex) is a synthetic long acting lutenising hormone releasing hormone (LHRH) agonist which has been shown to cause tumour shrinkage in premenopausal women with breast carcinoma (Williams *et al.*, 1986) and to a lesser extent in postmenopausal women (Harris *et al.*, 1989). This agent has been shown to suppress circulating levels of lutenising hormone, follicle stimulating hormone, testosterone, androstenedione and oestradiol in postmenopausal women receiving the drug by monthly depot subcutaneous (sc) injections (Dowsett *et al.*, 1988). Whilst this may not be relevant in oophorectomised women, it has been shown that women with epithelial ovarian cancer have higher levels of oestradiol than postmenopausal controls (Mählck *et al.*, 1988). It may therefore be postulated that oestradiol could increase growth of epithelial ovarian cancer cells by means of an autocrine loop. Therefore goserelin might be expected to exert its antiproliferative effect in epithelial ovarian cancer either due to a direct action on tumour cells via GnRH receptors, and/or secondary to its ability to lower circulating oestrogen and androgen levels by suppression of secretion by ovarian tumour cells. In order to test this hypothesis we have conducted a phase II study of goserelin in women with epithelial ovarian cancer.

Correspondence: B.M.J. Cantwell.
Received 31 July 1991; and in revised form 28 November 1991.

### Patients and methods

Women with histologically proven epithelial ovarian cancer who had relapsed following at least one trial of chemotherapy or who were considered too ill to receive chemotherapy for their disease were studied. There was no upper age limit. The details of these patients are shown in Table I. All patients had disease that was evaluable clinically or by ultrasound or computerised tomography (CT). Only symptomatic patients were treated. Twenty nine of the 30 had received prior chemotherapy. In all of these 29 patients chemotherapy had contained a platinum agent (either cisplatin or carboplatin) as part of their regimen. Eight patients had received prior endocrine therapy consisting of either tamoxifen or megesterol acetate, and three patients had received prior radiotherapy. One patient aged 90 years was treated with goserelin as first line therapy because of her age and poor performance status. The median interval between the start of first line chemotherapy, in the 29 patients, and disease progression was 52 (range, 0–285) weeks.

Patients were treated with monthly goserelin (Zoladex, ICI, Alderley Edge) 3.6 mg sc, after local infiltration of the site with lignocaine. Treatment was continued until disease progression became apparent.

At each monthly treatment patients were evaluated for toxicity and response status. Disease status was evaluated by clinical examination and sequential ultrasound or CT scanning where relevant. Standard WHO criteria were used to assess response and progression (WHO, 1979). Survival was defined as the period from diagnosis to death and response duration as the interval from response to disease progression.

### Results

Patients received a median of two cycles (range, 1–11) of goserelin. There were no complete responders but two (6.7%) patients achieved a partial remission lasting 40 and 105 weeks respectively. One patient with a poorly differentiated serous cystadenocarcinoma with an enlarged supraclavicular node and involved paraaortic nodes had progressed after three courses of carboplatin and subsequently responded to treatment with goserelin. The second responder who also had a poorly differentiated serous cystadenocarcinoma had

**Table I** Characteristics of 30 patients with epithelial ovarian cancer treated with goserelin

| | |
|---|---|
| *Median age (years)* | 57.5 (38–90) |
| *Median progression free interval (weeks)*[a] | 52 (0–285) |
| *ECOG performance status* | 2 (0–2) |
| *Histology* | |
| Serous | 14 |
| Mucinous | 4 |
| Clear cell | 1 |
| Endometroid | 1 |
| Mixed | 1 |
| Undifferentiated | 7 |
| Borderline | 2 |
| *Grade* | |
| Well differentiated | 2 |
| Moderately differentiated | 4 |
| Poorly differentiated | 18 |
| Undifferentiated | 1 |
| Borderline | 2 |
| Not classified | 3 |
| *Prior therapy* | |
| Median number (range) of prior therapies | 3 (0–6) |
| Chemotherapy | 29 |
| Chemotherapy (platinum based) | 29 |
| Hormone therapy | 8 |
| Radiotherapy | 3 |
| None | 1 |
| *Sites of disease* | |
| Abdomen (including paraortic nodes) | 22 |
| Pelvis | 17 |
| Liver | 6 |
| Lung | 2 |
| Supraclavicular nodes | 3 |
| Skin | 3 |
| Ascites | 10 |
| Pleural effusions | 3 |

[a]Progression free interval refers to the time following starting the first chemotherapy regimen and the first relapse.

initially been treated with carboplatin and had disease stabilisation for 7 months. On progression she had received a second course of carboplatin, but had progressed after only two cycles. After this she had progressed through treatment with high dose tamoxifen and etoposide and subsequently mitomycin C and 5 fluorouracil. Following this she underwent debulking surgery which left her with a residual pelvic mass. This responded to goserelin treatment. Five patients had disease stabilisation lasting 25, 35, 40, 66 and 70 weeks respectively. Twenty three patients had progressive disease and this included five early deaths. These five deaths occurred before a second cycle of goserelin was given. Even if the early deaths are counted as due to disease progression in the analysis, then seven of the 30 patients (23%, 95% confidence interval 10–42%) had absence of disease progression for 6 months or longer. There were no significant or unexpected toxicities, other than the inconvenience for patients attending hospital for subcutaneous injections, and the minor irritation of the injection site seen in some patients. The median survival following commencement of goserelin treatment was 26 (range, 1–192) weeks. No subjective or objective toxicity was witnessed.

### Discussion

The fact that ovarian tumours possess sex hormone receptors has meant that endocrine therapy for recurrent disease has been regarded as an attractive alternative to conventional cytotoxic chemotherapy. Most efforts at endocrine therapy in epithelial ovarian cancer have concentrated on the use of either progestagens or the antioestrogen tamoxifen. Several progestagens have been used including 17-a-hydroxyprogesterone, 17-a-hydroxy-19-norprogesterone-17-n-caproate, 6, 17-a-dimethyl-6-dehydroprogesterone, medroxyprogesterone acetate, and megesterol acetate. Unfortunately response rates with these agents have been disappointingly low ranging from 10–15% (Slotman & Rao, 1988). The other most widely used hormonal agent in the treatment of epithelial ovarian cancer has been the antioestrogen tamoxifen which has produced objective response rates from 0–10% (Slotman & Rao, 1988). Despite the expression of oestrogen receptors in a sizable proportion of human ovarian carcinomas, and a positive relationship between tumour volume and plasma oestradiol levels (Mählck *et al.*, 1988), the poor clinical results with endocrine manipulations designed to perturb oestrogen regulated tumour growth, suggest that this mechanism of growth control is of only minor clinical importance.

We have used an LHRH agonist as it might be expected to reduce tumour cell proliferation by both a direct action and by reducing circulating levels of oestradiol, androstenedione and testosterone. To date there have been only two studies with LHRH agonists used in the treatment of advanced epithelial ovarian cancer. Both of these studies have used leuprolide. Kavanagh *et al.* (1989) achieved a 17% partial response rate in 23 patients whom had been previously treated with chemotherapy. Additionally a further two patients had disease stabilisation. In a second study by Bruckner and Motwani (1989) five patients were treated with leuprolide and megesterol acetate and four of these patients achieved an objective response.

Our phase II study of goserelin in advanced epithelial ovarian cancer was the first to use goserelin. Our objective response rate was only 6.7% (2/30) but most patients had very advanced cancers and were heavily pre-treated. Further more we included two patients with borderline malignancies (pseudomyxoma peritoneii) whom are generally refractory to most cytotoxic or endocrine therapies. Overall seven (23%) patients had absence of disease progression for 6 months or more. In advanced breast cancer absence of disease progression i.e. disease stabilisation for 5 months or more following therapy confers the same survival benefit as partial response to therapy (Howell *et al.*, 1988). Thus the disease stabilisation seen in our patients may confer some survival advantage. LHRH agonists should be evaluated earlier in the management of women with epithelial ovarian cancer, perhaps at first relapse or in patients resistant to first line conventional cytotoxic chemotherapy.

If our results are validated, then future studies could address the combination of goserelin with antiandrogens which might provide better growth inhibition, targeting both putative autocrine loop elements (LHRH receptors) and an androgen mediated endocrine drive. Androgens may be of greater importance in epithelial ovarian cancer than oestrogens since as many as 88% of these tumours were found to express androgen receptors (Rao *et al.*, 1990).

### References

BLACKLEDGE, G., LAWTON, F., REDMAN, C. & KELLY, K. (1989). Response of patients in phase II studies of chemotherapy in ovarian cancer: implications for patient treatment and the design of phase II trials. *Br. J. Cancer*, **59**, 650.

BRUCKNER, H.W. & MOTWANI, B.T. (1989). Treatment of advanced refractory ovarian carcinoma with a gonadotrophin-releasing hormone analogue. *Am. J. Obstet. Gynecol.*, **161**, 1216.

DOWSETT, M., CANTWELL, B., LAL, A., JEFFCOATE, S.L. & HARRIS, A.L. (1988). Suppression of Postmenopausal steroidogenesis with luteinizing hormone-releasing hormone agonist goserelin. *J. Clin. Endocrinol. Metab.*, **66**, 672.

EMONS, G., PAHWA, G.S., BRACK, C., STURM, R., OBERHEUSER, F. & KNUPPEN, R. (1989). Gonadotrophin releasing hormone binding sites in human epithelial ovarian carcinomata. *Eur. J. Cancer Clin. Oncol.*, **25**, 215.

HARRIS, A.L., CARMICHAEL, J., CANTWELL, B.M.J. & DOWSETT, M. (1989). Zoladex: endocrine and therapeutic effects in post-menopausal breast cancer. *Br. J. Cancer*, **59**, 97.

HOWELL, A., MACKINTOSH, J., JONES, M., REDFORD, J., WAGSTAFF, J. & SELLWOOD, R.A. (1988). The definition of the 'No Change' category in patients treated with endocrine therapy and chemotherapy for advanced carcinoma of the breast. *Eur. J. Cancer Clin. Oncol.*, **24**, 1567.

KAVANAGH, J.J., ROBERTS, W., TOWNSEND, P. & HEWITT, S. (1989). Leuprolide acetate in the treatment of refractory or persistent epithelial ovarian cancer. *J. Clin. Oncol.*, **7**, 115.

LANGDON, S.P., HAWKES, M.M., LAWRIE, S.S. & 6 others (1990). Oestrogen receptor expression and the effects of oestrogen and tamoxifen on the growth of human ovarian carcinoma cell lines. *Br. J. Cancer*, **62**, 213.

MÄHLCK, C.-G., BÄCKSTRÖM, T. & KJELLGREN, O. (1988). Plasma level of oestradiol in patients with ovarian malignant tumours. *Gynecol. Oncol.*, **30**, 313.

RAO, B.R., SLOTMAN, B.J., GELDOF, A.A. & DINJENS, W.N.M. (1990). Correlation between tumour histology, steroid receptor status, and adenosine deaminase complexing protein immunoreactivity in ovarian cancer. *Int. J. Gynecol. Pathol.*, **9**, 47.

SLOTMAN, B.J. & RAO, B.R. (1988). Ovarian Cancer (Review). *Anticancer Res.*, **8**, 417.

WHARTON, J.T., EDWARDS, C.L. & RUTLEDGE, F.N. (1984). Long term survival after chemotherapy for advanced epithelial ovarian carcinoma: initial experience using a platinum based combination. *Cancer*, **49**, 1778.

WILLIAMS, M.R., WALKER, K.J., TURKES, A., BLAMEY, R.W. & NICHOLSON, R.I. (1986). The use of an LH-RH agonist (ICI 118630, Zoladex) in advanced premenopausal breast cancer. *Br. J. Cancer*, **53**, 629.

WORLD HEALTH ORGANIZATION (1979). Handbook for reporting results of Cancer Treatment. *WHO Offset Publ. no.* **48**. WHO: Geneva.


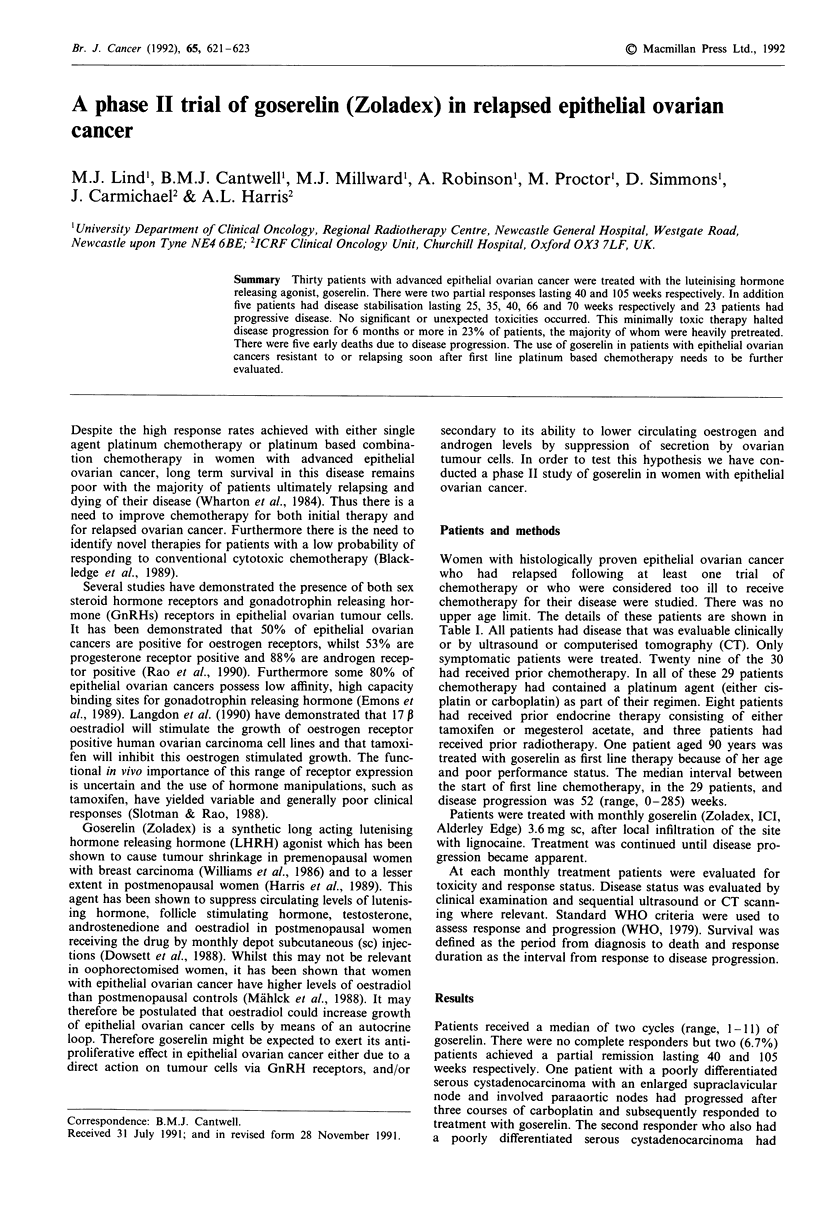

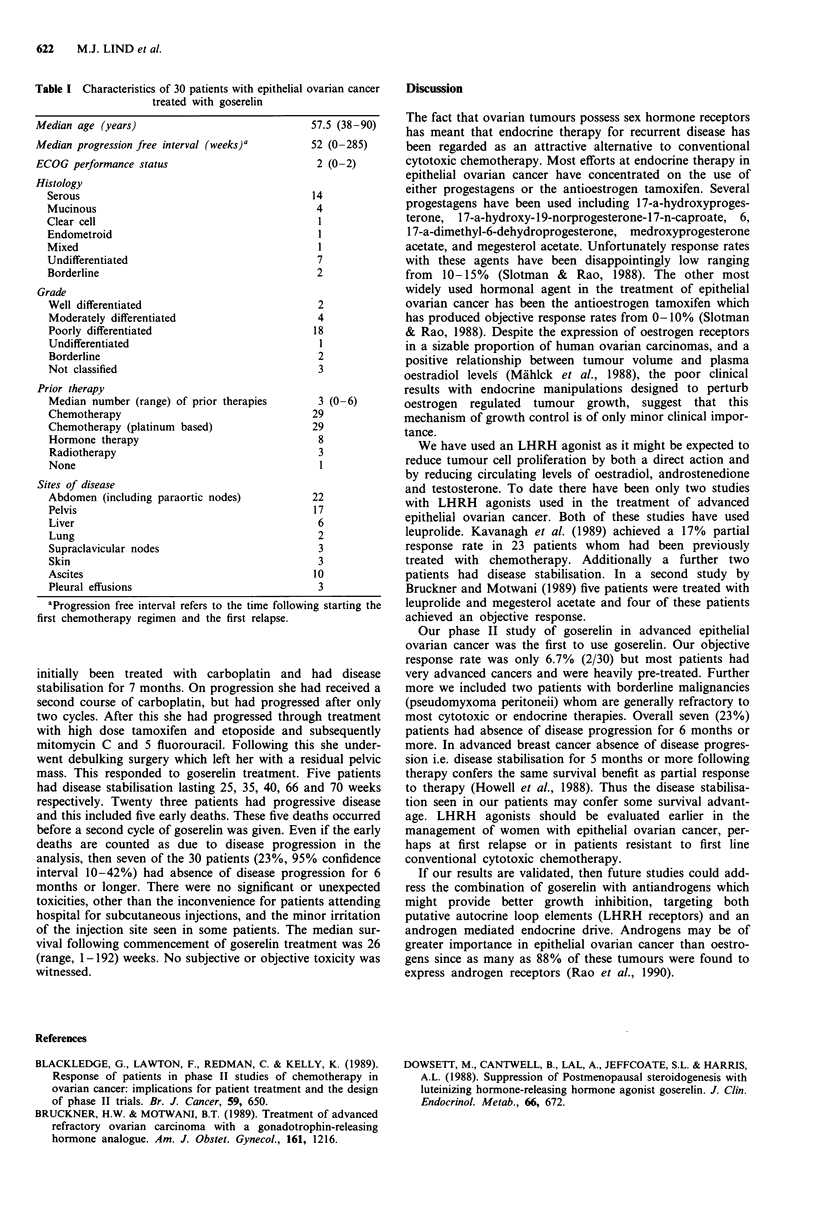

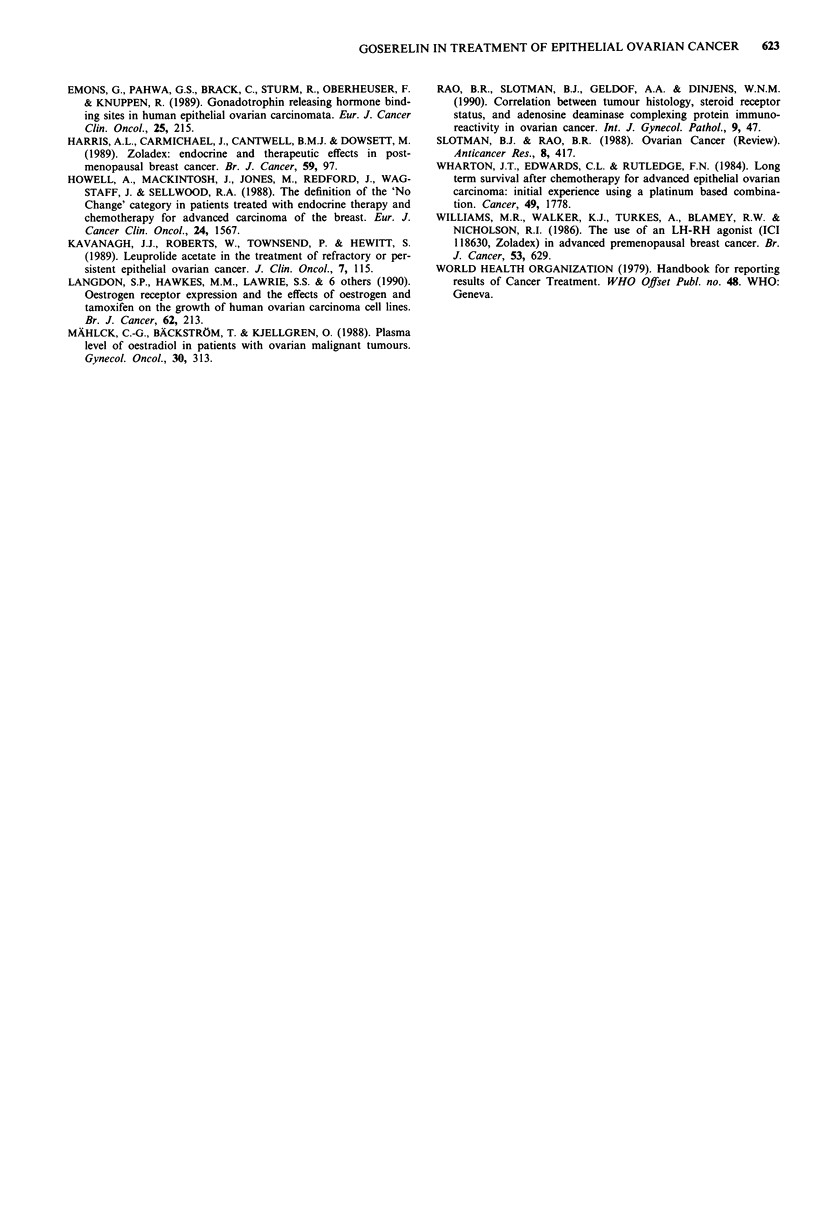


## References

[OCR_00272] Blackledge G., Lawton F., Redman C., Kelly K. (1989). Response of patients in phase II studies of chemotherapy in ovarian cancer: implications for patient treatment and the design of phase II trials.. Br J Cancer.

[OCR_00278] Bruckner H. W., Motwani B. T. (1989). Treatment of advanced refractory ovarian carcinoma with a gonadotropin-releasing hormone analogue.. Am J Obstet Gynecol.

[OCR_00283] Dowsett M., Cantwell B., Lal A., Jeffcoate S. L., Harris A. L. (1988). Suppression of postmenopausal ovarian steroidogenesis with the luteinizing hormone-releasing hormone agonist goserelin.. J Clin Endocrinol Metab.

[OCR_00291] Emons G., Pahwa G. S., Brack C., Sturm R., Oberheuser F., Knuppen R. (1989). Gonadotropin releasing hormone binding sites in human epithelial ovarian carcinomata.. Eur J Cancer Clin Oncol.

[OCR_00297] Harris A. L., Carmichael J., Cantwell B. M., Dowsett M. (1989). Zoladex: endocrine and therapeutic effects in post-menopausal breast cancer.. Br J Cancer.

[OCR_00304] Howell A., Mackintosh J., Jones M., Redford J., Wagstaff J., Sellwood R. A. (1988). The definition of the 'no change' category in patients treated with endocrine therapy and chemotherapy for advanced carcinoma of the breast.. Eur J Cancer Clin Oncol.

[OCR_00309] Kavanagh J. J., Roberts W., Townsend P., Hewitt S. (1989). Leuprolide acetate in the treatment of refractory or persistent epithelial ovarian cancer.. J Clin Oncol.

[OCR_00314] Langdon S. P., Hawkes M. M., Lawrie S. S., Hawkins R. A., Tesdale A. L., Crew A. J., Miller W. R., Smyth J. F. (1990). Oestrogen receptor expression and the effects of oestrogen and tamoxifen on the growth of human ovarian carcinoma cell lines.. Br J Cancer.

[OCR_00320] Mählck C. G., Bäckström T., Kjellgren O. (1988). Plasma level of estradiol in patients with ovarian malignant tumors.. Gynecol Oncol.

[OCR_00325] Rao B. R., Slotman B. J., Geldof A. A., Dinjens W. N. (1990). Correlation between tumor histology, steroid receptor status, and adenosine deaminase complexing protein immunoreactivity in ovarian cancer.. Int J Gynecol Pathol.

[OCR_00331] Slotman B. J., Rao B. R. (1988). Ovarian cancer (review). Etiology, diagnosis, prognosis, surgery, radiotherapy, chemotherapy and endocrine therapy.. Anticancer Res.

[OCR_00341] Williams M. R., Walker K. J., Turkes A., Blamey R. W., Nicholson R. I. (1986). The use of an LH-RH agonist (ICI 118630, Zoladex) in advanced premenopausal breast cancer.. Br J Cancer.

